# Indents

**Published:** 1923-08

**Authors:** 


### INDENTS

**☞Brief Articles of Technical or Scientific Value will receive notice and comment. Contributions invited.**

AN UNUSUAL CASE. A remarkable case is reported by Dr. Menifee Howard, in the Journal of the American Dental Association. The patient was a girl of twenty-one. She was suffering from severe malformation of the mouth and teeth, which had affected her whole outlook to such an extent that she appeared to have lost all interest in life altogether. Dr. Howard describes the condition of the patient when he first saw her as follows:

"The patient in this case was only twenty-one but she looked every bit of forty years old. She weighed eighty-two pounds, was married, and the mother of a three-year-old girl. Her face and features were not good to look upon, rather repulsive.

After talking to her, I soon made up my mind, owing to her dress, personal appearance, etc., that the charms of life had little or no attraction to her. Nature had been a little unkind (really nature had almost ruined her). She seemed to be bordering on a general breakdown physically, very nervous, tiring easily and no resistance. From the mental point of view she was down in the depths, taking little interest in anything and bemoaning her fate.

All her plight seemed to center around a malformation of the mouth, evidenced by a protrusion of the teeth and lips, much like pigs' teeth. Owing to the extensive protrusion, she could not close her lips, and had not been able to close them at any time in previous years that she could remember. This caused a dry, uncomfortable condition of the mouth and one predisposed to infection.

Upon examining the patient's mouth, I saw the most unusual condition I had ever witnessed. The arches were constricted and the gums hypertrophied out of all proportion. The teeth on both the upper and lower jaws were scattered about the mouth, lying in almost all positions except upside down. The lower teeth on each side, actually extended horizontally across the mouth, interfering with the normal position of the tongue. There were thirty-two teeth in the mouth, but no tooth was touching the other in its position in the jaw, and when the jaws were in occlusion not more than two or three teeth touched each other. The abnormal hypertrophied gum tissue covered most of the crown surface so that only the tip end of most teeth were exposed. Because of this condition mastication was accomplished mostly upon the gums. By pressing the finger on the gums almost any place in the mouth several drops of pus could be expressed.

In the patient's endeavor to close the lips, pressure would be made on the gums so when she opened the lips in talking or smiling little yellow drops of pus would exude which was very disagreeable to look upon.

### HEREDITARY

This is a picture of a very miserable woman, and her misery appears to have been due entirely to dental disorder. Examination of her child revealed a tendency to hypertrophy of the gums similar to the condition found in the mother. The father of the patient had died at the age of twenty-eight, so the information obtained in regard to him was meager. It was stated, however, that he was a small, ill-nourished man, and that his gums revealed the same condition of hypertrophy as the daughter. No information as regards the mother of the patient is obtainable.

These facts, however, are suggestive. It seems clear that heredity played a very definite part in the production of the remarkable condition present in this patient. Hypertrophy of the gums is frequently hereditary, though it is rare to find so extreme a condition as that above described.

An examination by a general physician revealed little abnormal apart from the mouth. A definite anemia was present, and there were the scars of operations upon the right upper arm. The history showed that these were the result of operations performed for the relief of a streptococcal cellulitis and osteomyelitis five years previously. Radiographic examination revealed the presence of many extensive areas of infection in the alveolar bone.

### THE TREATMENT

It was clear that the chronic general periodontitis had progressed far beyond the stage where treatment, other than extraction, was of any avail, nor could orthodontists hold out any hope of relieving the irregularity. There remained, therefore, two courses open, viz.: to leave the patient *in statu quo,* to work out a miserable existence until released by death, or to devise some very drastic form of operative treatment, which though naturally experimental, yet held out some hope of improvement. Dr. Howard advised the second alternative, and the patient seized upon the suggestion with enthusiasm. The ultimate result more than justified this decision.

The teeth were all extracted, the hypertrophied gum removed, and the alveolus pared down. Marked improvement in the general condition of the patient was at once noticed. After a short interval, dentures were inserted.

The change in the patient's appearance, as shown by photographs was very marked. She appeared quite a different woman. By the operation, a slatternly, miserable, repulsive object was transformed into a healthy, cheerful, pleasant-looking girl, full of energy and *joie de vivre.*

Such opportunities rarely come the way of the dental specialist, but it is to be hoped that on those rare occasions when such cases do present themselves, the dentist will be found ready and able to do what is required of him.

---

The First Permanent Molar. In the *Dental Summary* Dr. Frederick Noyes makes a strong appeal for the preservation of the first permanent molars wherever possible. He believes that the removal of these teeth is invariably followed by irreparable damage to the denture as a whole, and to the harmony of the face, and strongly deprecates their extraction except in extreme cases, where they are so far diseased as to be unsaveable.

The following are some fundamental facts concerning the first permanent molars upon which Dr. Noyes lays special stress:

"1. They erupt back of the second temporary molars while the temporary dentition is still intact. For this reason, they should be guided into normal relation, and they maintain the normal relation of the mandible to the maxilla during the period of the loss of the temporary denture and its replacement by the permanent one.

2. In all of the periods, from six to eight years of age, they are the largest, strongest and most efficient portion of the masticating apparatus. They are firm in their attachment, while the temporary teeth are being loosened and replaced.

3. They determine the balance of forces between the muscles attached to the anterior and posterior portions of the mandible which result in the normal form of this and related bones.

4. Their loss always affects the relation of all of the teeth to the skull, reducing the efficiency of the denture as a masticating machine and marring the symmetry of the features.

The first molars should, therefore, be most carefully guarded, to preserve them from destruction by caries, to which they are more liable than any other tooth. Their loss destroys the masticatory efficiency of the denture during the most important developmental period, and causes more or less deformity.

Their malrelation always causes malocclusion in the denture and deformity of the face. Their eruption should be watched; if necessary they should be guided into normal relation, so that the normal distribution of forces may allow development to proceed toward the normal instead of away from it."

### RESULTS OF REMOVAL OF FIRST PERMANENT MOLARS

Dr. Noyes proceeds to quote cases to show the serious results that may follow extraction of these teeth. He has naturally chosen rather extreme cases, which well illustrate his point of view. Without in any way denying the importance of the first molars, it may be suggested that many cases could be brought forward in which one or more of these teeth have been extracted, while the patients still possess thoroughly useful and functional masticatory apparatus, and reasonably pleasant and harmonious faces.

In cases of general crowding, where all the teeth are free from caries, there are few who would not preserve the first molars at the expense of premolars; but there are many who would hesitate to sacrifice healthy premolars in order to retain first molars that have been attacked by caries.

The difference of opinion is only one of degree and chiefly depends upon the answer to the question: How far diseased must a first permanent molar be before it should be extracted, in preference to a healthy premolar? Upon such a question it is hardly likely that there will ever be complete unanimity among dentists. Albeit, the more often such matters are freely discussed the more shall we all add to our knowledge.

